# CircRNA circ_0092314 Induces Epithelial-Mesenchymal Transition of Pancreatic Cancer Cells *via* Elevating the Expression of S100P by Sponging miR-671

**DOI:** 10.3389/fonc.2021.675442

**Published:** 2021-03-25

**Authors:** Qian Shen, Gang Zheng, Yi Zhou, Jin Tong, Sanpeng Xu, Hui Gao, Xiaofan Zhang, Qiang Fu

**Affiliations:** ^1^ Department of Oncology, Tongji Hospital, Tongji Medical College, Huazhong University of Science and Technology, Wuhan, China; ^2^ Department of General Surgery, The Fifth Hospital of Wuhan, Wuhan, China; ^3^ Department of Gastrointestinal Surgery, Tongji Hospital, Tongji Medical College, Huazhong University of Science and Technology, Wuhan, China; ^4^ Department of PICC, Tongji Hospital, Tongji Medical College, Huazhong University of Science and Technology, Wuhan, China; ^5^ Department of Pathology, Tongji Hospital, Tongji Medical College, Huazhong University of Science and Technology, Wuhan, China; ^6^ Department of Clinical Nutrition, Tongji Hospital, Tongji Medical College, Huazhong University of Science and Technology, Wuhan, China; ^7^ Department of Neurology, Tongji Hospital, Tongji Medical College, Huazhong University of Science and Technology, Wuhan, China

**Keywords:** circular RNA, circ_0092314, microRNA, emt, miR-671, s100p, pancreatic cancer

## Abstract

**Background:**

Circular RNAs (circRNAs) is a novel class of non-coding RNAs that regulate gene expression during cancer progression. Circ_0092314 is a newly discovered circRNA that was upregulated in pancreatic cancer (PAAD) tissues. However, the detailed functions and underlying mechanisms of circ_0092314 in PAAD cells remain unclear.

**Methods:**

We first determined the expression of circ_0092314 in PAAD and normal tissues and further investigated the functional roles of circ_0092314 in regulating epithelial-mesenchymal transition (EMT) of PAAD cells. We also assessed the regulatory action of circ_0092314 on the microRNA-671 (miR-671) and its target S100P.

**Results:**

Circ_0092314 was markedly upregulated in PAAD tissues and cells, and its overexpression was closely correlated with worse prognosis of PAAD patients. Functionally, circ_0092314 promotes proliferation, invasion and EMT *in vitro* and tumor growth *in vivo*. Mechanistically, we demonstrated that circ_0092314 directly binds to miR-671 and relieve its suppression of the downstream target S100P, which induces EMT and activates the AKT signaling pathway. The tumor-promoting effects caused by overexpression of circ_0092314 could be revered by re-expression of miR-671 in PAAD cells.

**Conclusions:**

Overall, our study demonstrates that circ_0092314 exerts critical roles in promoting the EMT features of PAAD cells, and provides insight into how elevated expression of circ_0092314 might influence PAAD progression.

## Background

Pancreatic ductal adenocarcinoma (PAAD) is a highly aggressive cancer type, being characterized by high rates of metastasis and resistance to chemotherapy (1). Epithelial-mesenchymal transition (EMT) is a phenomenon in which epithelial cells lose contact between neighboring cells and acquire migratory mesenchymal phenotypes (2). Hallmarks of EMT include loss of epithelial markers, such as E-cadherin, and gain of mesenchymal markers, such as Vimentin (2). Cancer stem cells (CSCs) are subpopulations of cancer cells that show stem cell characteristics and influence tumorigenesis, metastasis and chemoresistance (3). The pancreatic cancer stem cells express cell surface markers, such as CD44 and CD133 (3). It is considered that cancer cells can enter the CSC state *via* activation of the EMT program (4).

S100P protein, a small isoform of the S100 protein family, is frequently overexpressed in human tumors including PAAD (5). S100P was shown to increase the migratory and invasive capabilities of PAAD cells (6). Although a previous study in pancreatic cancer has shown that S100P contributes to the aggressive nature and EMT features of colon cancer cells (7), the association of S100P expression with EMT induction in PAAD needs further exploration.

MicroRNAs (miRNAs) are endogenous, small non-coding RNAs that mediate gene expression through interaction with 3′-untranslated regions (3′-UTRs) of mRNAs (8). Circular RNAs (circRNAs) are an emerging subgroup of non-coding RNAs that are more stable than other RNAs and can act as competing endogenous RNAs (ceRNAs) to sponge miRNAs, thus indirectly regulating the expression of target genes of miRNAs (9, 10). Growing evidence has shown that the dysregulation of circRNAs is associated with the initiation and development of many cancers, including PAAD (9–11). For example, circ_0092314 is a newly discovered circRNA that was upregulated in PAAD tissues (12). However, the detailed functions and the molecular mechanism underlying the role of circ_0092314 in PAAD cells are not fully understood.

Here, we identified a novel role of circ_0092314 in regulating EMT and invasion of PAAD cells. Circ_0092314 induces EMT of PAAD cells by competing for miR-671 to subsequently increase the expression of its downstream target of S100P. Our results suggest that circ_0092314 would be a promising target for PAAD therapy.

## Materials and Methods

### Clinical Tissue Samples

This research has been approved by the Helsinki declaration and approved by the Research Ethics Committee of Tongji Hospital, Tongji Medical College, Huazhong University of Science and Technology, China. Each patient signed the informed consent. Forty PAAD tissues and adjacent normal tissues were collected during surgical excision. Tissues were immediately frozen in liquid nitrogen and stored at -80°C.

### Cell Lines, Cell Culture, and Transfection

Four PAAD cell lines (AsPC-1, BxPC-3, SW-1990 and PaCa-2) and a human pancreatic duct epithelial cell line HPDE6-C7 were provided by the American Type Culture Collection (Manassas, VA, USA). All cells were cultured in RPMI1640 medium (Thermo Fisher Scientific, Waltham, MA, USA) supplemented with 10% fetal bovine serum (FBS, Thermo Fisher Scientific) in a 37°C, 5% CO2 humidified atmosphere.

Transient transfection was done using Lipofectamine 2000 reagent (Invitrogen, Carlsbad, CA, USA) in accordance with the manufacturer’s instructions. The overexpression vectors for circ_0092314 and S100P were synthesized by Geneseed (Guangzhou, China). SiRNAs targeting circ_0092314 and S100P, as well as miR-671 mimics and miR-671 inhibitors, were purchased from Genechem (Shanghai, China).

### Quantitative Real-Time PCR (qRT-PCR)

Total RNA was extracted from tissues or cells by TRIzol reagent (Invitrogen). Total RNA from PAAD cells was divided into two groups and one group was treated with 3 U/µg RNase R (Geneseed, Guangzhou, China) at 37 °C, for 30 min according to the manufacturer’s instructions. The nuclear and cytoplasmic RNA fractionation was isolated with PARIS Kit (Invitrogen). Reverse transcription was performed using the PrimeScript RT Master Mix (Takara, Dalian, China). Quantitation of circ_0092314 and S100P mRNA was performed using a SYBR PCR kit (Takara), as previously described (13). The expression level of miR-671 was evaluated using the NCode miRNA qRT-PCR analysis (Invitrogen). The levels of circ_0092314 and S100P were normalized to the control GAPDH. The expression of miR-671 was normalized to the control U6. All primers used in this study were obtained from Genechem (Shanghai, China).

### Western Blotting

Total protein was extracted using a RIPA buffer (Beyotime, Beijing, China). SDS-polyacrylamide gel electrophoresis was performed using an equal amount of total protein. Then, the protein was transferred onto PVDF membranes (Millipore, Bedford, MA, USA). Next, membranes were incubated with the primary antibodies: E-cadherin (Cell Signaling, MA, USA; 1:1000), Vimentin (Cell Signaling; 1:1000), S100P (Cell Signaling; 1:1000), AKT (Cell Signaling; 1:1000), p-AKT (Cell Signaling; 1:1000) and β-actin (Cell Signaling; 1:5000). Next day, the secondary antibodies were added and each protein band was detected by the ECL detection system (Amersham Biosciences, Buckinghamshire, UK).

### Cell Counting Kit-8 Assay

The proliferation rate of PAAD cells were assessed by Cell Counting Kit-8 assay (CCK-8, Dojindo, Kumamoto, Japan) according to the manufacturer**’**s instructions. 5 × 10^3^ cells were seeded into 96-well plates. At 24, 48, 72 and 96 h after transfection, 10 μl of CCK-8 reagent was added to each well and incubated for 2 h. The absorbance at 450 nm was measured using an automatic microplate reader (BioTek, VT, USA).

### Transwell Invasion Assay

Cell invasion assay was performed as previously reported (14). We placed 750 μl medium containing 10% FBS in the bottom chamber, and then seeded PAAD cells into the upper chamber for invasion assays (8-μm pore size, Corning, CA, USA) in 500 μl serum-free media. After 24 h, the invaded cells were stained with Giemsa (Sigma, St. Louis, MO, USA) for 15 minutes. Finally, the number of cells was counted using an Olympus microscope.

### Tumor Sphere Formation Assay

Single cells were seeded in 24-well ultra-low attachment plates (Corning, Acton, MA) containing serum-free medium supplemented with B27 (1:50; Invitrogen, Carlsbad, CA, USA), 20 ng/ml basic FGF (BD Biosciences, CA, USA) and 20 ng/ml EGF (Sigma, St. Louis, MO, USA). The cultures were fed with fresh serum-free medium and growth factors every other day. After 14 days of incubation, images of cells were captured and tumor spheres with a diameter more than 50 μm were counted using Image J software.

### *In Vivo* Tumor Formation Assay

All animal experiments were performed according to protocols approved by the Institutional Animal Care and Use Committee of Tongji Hospital, Tongji Medical College, Huazhong University of Science and Technology, China. Four-week-old Nude mice (*n* = 6 per group) were purchased from Beijing HFK Bioscience (Beijing, China). PAAD cells transfected as indicated were transplanted subcutaneously into the right flank of nude mice. The tumor volumes were measured every week and calculated as length (mm) × width^2^ (mm^2^) × 0.5. After 3 weeks after injection, the mice were sacrificed. The tumor were carefully removed, photographed and weighed.

### Luciferase Reporter Assay

A circ_0092314 fragment or a 3′-UTR sequence of S100P was inserted into a luciferase reporter plasmid (Ribobio, Guangzhou, China). Mutagenesis was performed using a QuickChange Site-Directed Mutagenesis kit (Stratagene, CA, USA). PAAD cells were transiently co-transfected with luciferase reporter plasmid with miR-671 mimic (or miR-671 inhibitor) using Lipofectamine 2000 Reagent (Invitrogen), along with the Renilla luciferase plasmid pRL-CMV (Promega, WI, USA). After 48 h of transfection, the cells were harvested and subjected to a Dual-Luciferase Reporter Assay system (Promega). The relative luciferase activities were normalized with the Renilla luciferase activities.

### RNA Immunoprecipitation (RIP) Assay

RIP assay was conducted using a Magna RIP RNA-Binding Protein Immunoprecipitation Kit (Millipore, Billerica, MA, USA). PAAD cells were lysed with a RIP-lysis buffer and cell lysates were incubated with magnetic beads conjugated with anti-Ago2 antibody (Millipore) or control anti-IgG antibody (Millipore). After incubating with proteinase K, the immunoprecipitated RNAs were extracted and subjected to the qRT-PCR analysis.

### Statistical Analysis

All results were representative of at least three independent experiments. All data were shown as the mean ± standard deviation, and experimental data were evaluated using the Student**’**s *t*-tests, one-way ANOVA tests and Wilcoxon signed-rank tests. The statistical analysis was performed using SPSS 25.0 statistical software package. * *P* < 0.05 was considered statistically significant.

## Results

### Circ_0092314 Is Highly Expressed in PAAD Tissues and Cells

**Figure 1:**
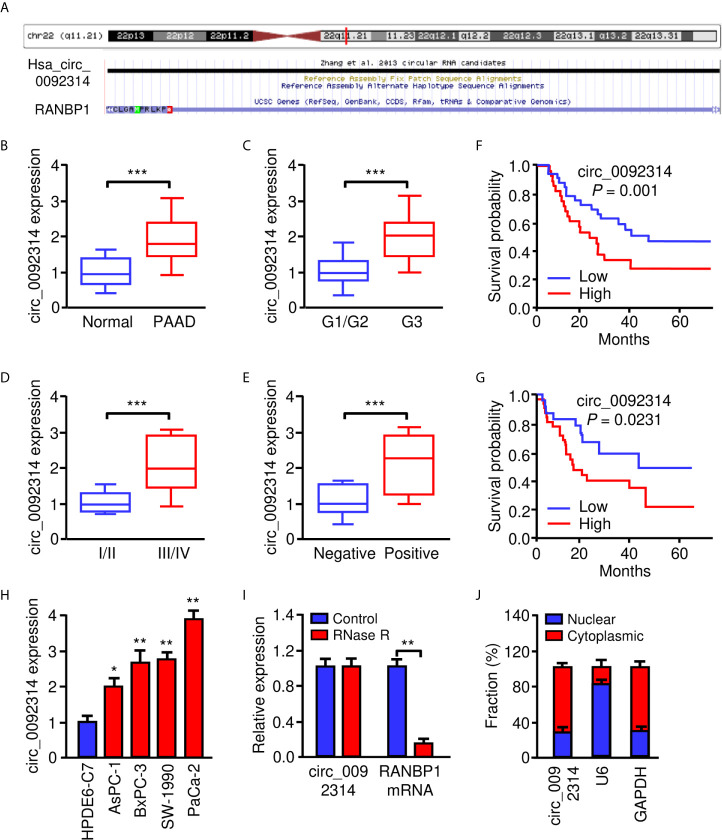
Circ_0092314 is highly expressed in PAAD tissues and cells. **(A)** Human *RANBP1* gene was identified as the host gene of circ_0092314 by browsing circBase database. **(B)** The expression of circ_0092314 in PAAD tissues (n = 40) and their adjacent normal pancreatic tissues (n = 40) was determined using qRT-PCR assay. **(C)** The expression of circ_0092314 in well/moderate and poor differentiated PAAD tissues from patients. **(D)** The expression of circ_0092314 in PAAD tissues from patients with stages I and II, or stage III and IV disease. **(E)** The expression of circ_0092314 in PAAD tissues from patients with (or without) lymph node metastasis. **(F, G)** Kaplan-Meier survival analysis showed that PAAD patients with high circ_0092314 expression have a lower overall survival **(F)** and disease-free survival **(G)** than those with low circ_0092314 expression. **(H)** qRT-PCR analysis of circ_0092314 expression in PAAD cell lines and HPDE6-C7 cells. **(I)** The expression of circ_0092314 and RANBP1 mRNA in PaCa-2 cells was detected using qRT-PCR assay in the presence or absence of RNase R. **(J)** The nuclear-cytoplasmic fractionation assay showed that circ_0092314 was mainly located in the cytoplasm. *P < 0.05, **P < 0.01, ***P < 0.001.

Using the circBase database (http://www.circbase.org/), we found that circ_0092314 was produced from human *RANBP1* gene located at chr22: 20113099-20113439, and finally formed a circular transcript of 340 nt (**Figure 1A**). To investigate the biological role of circ_0092314 in PAAD, we first analyzed its expression in 40 pairs of PAAD specimens and adjacent normal tissues using qRT-PCR assay. The expression level of circ_0092314 was significantly upregulated in PAAD tissues compared with adjacent normal tissues (**Figure 1B**). Subsequently, we divided PAAD tissues as well/moderately and poorly differentiated groups, low TNM stages (I-II) and high TNM stages (III-IV) groups, as well as lymph node metastasis-negative and -positive groups. As shown in **Figures 1C, D, E**, the levels of circ_0092314 in the poorly differentiated group, high TNM stage group, or lymph node metastasis-positive group were significantly higher than those in the well/moderately group, low TNM stage or lymph node metastasis-negative group. Furthermore, the expression of circ_0092314 in PAAD tissues was divided into the low expression group and the high expression group based on the median values. Our Kaplan-Meier analysis showed that PAAD patients with high circ_0092314 expression had a significantly poorer overall survival and disease-free survival compared with patients with low circ_0092314 expression (**Figures 1F, G**).

Then, the expression of circ_0092314 in four PAAD cell lines (AsPC-1, BxPC-3, SW-1990 and PaCa-2) and human pancreatic duct epithelial cell line (HPDE6-C7) was assessed by qRT-PCR experiments. Increased expression of circ_0092314 was observed in all PAAD cell lines compared with HPDE6-C7 cells (**Figure 1H**). Among these cell lines, PaCa-2 cells expressed the highest level of circ_0092314, while AsPC-1 cells had the lowest level of circ_0092314 (**Figure 1G**). RNase R was administrated to the extracted RNA from PaCa-2 cells. We found that the circular form (circ_0092314) can be resistant to RNase R, whereas the linear form (RANBP1 mRNA) was significantly decayed (**Figure 1I**). In addition, qRT-PCR analysis of nuclear and cytoplasmic fractions of RNAs demonstrated that circ_0092314 was predominantly localized in the cytoplasm (**Figure 1J**). Taken together, our results suggest that circ_0092314 is an abundant and stable circRNA overexpressed in PAAD cells.

### Circ_0092314 Enhances the Proliferation of PAAD Cells *In Vitro* and *In Vivo*

**Figure 2:**
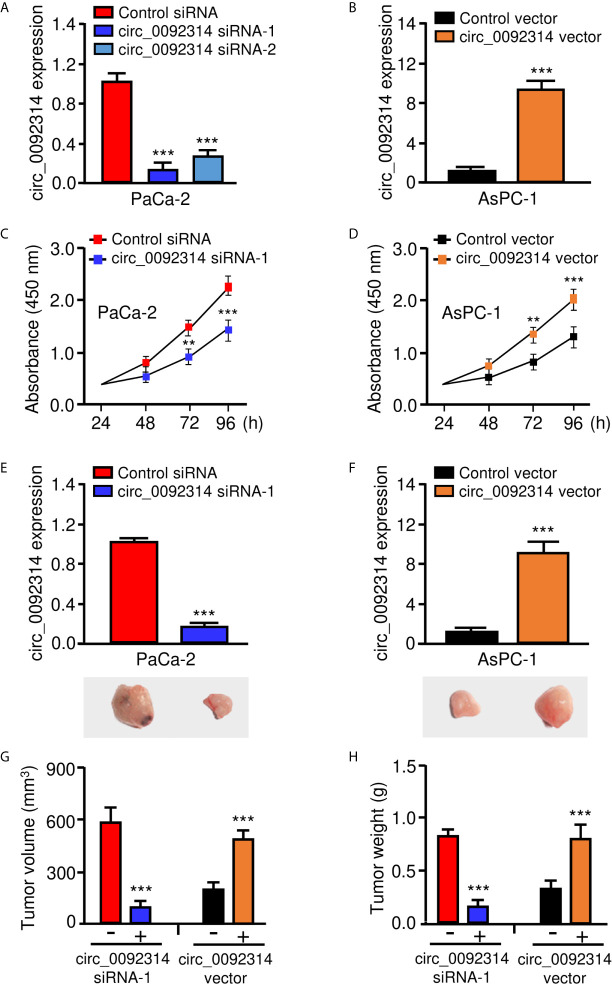
Circ_0092314 enhances the proliferation of PAAD cells *in vitro* and *in vivo*. **(A)** The expression of circ_0092314 in PaCa-2 cells transfected with siRNAs targeting circ_0092314 or control siRNA. **(B)** The expression of circ_0092314 in AsPC-1 cells transfected with circ_0092314 overexpression vector or control vector. **(C)** CCK-8 assays in PaCa-2 cells transfected with siRNAs targeting circ_0092314 or control siRNA. **(D)** CCK-8 assays in AsPC-1 cells transfected with circ_0092314 overexpression vector or the control vector. **(E, F)** The expression of circ_0092314 in PaCa-2 **(E)** and AsPC-1 **(F)** cells was examined using qRT-PCR assay. The lower panels showed the representative images of xenograft tumors in nude mice. **(G, H)** Tumor volume **(G)** and tumor weight **(H)** were measured from each group after 21 days. **P < 0.01, ***P < 0.001.

We determined the biological roles of circ_0092314 in PAAD cells. To this end, we transfected PaCa-2 cells with specific siRNAs targeting circ_0092314, and transfected AsPC-1 cells with overexpression plasmid of circ_0092314, respectively. Circ_0092314 expression was significantly silenced in PaCa-2 cells, whereas its expression was clearly increased in AsPC-1 cells following overexpression of circ_0092314 (**Figures 2A, B**). The proliferation of PaCa-2 cells was decreased by downregulation of circ_0092314, and overexpression of circ_0092314 promoted the proliferation of AsPC-1 cells (**Figures 2C, D**). To investigate the function of circ_0092314 *in vivo*, we established a nude mice xenograft model of PAAD by subcutaneous inoculation of PaCa-2 cells transfected with specific siRNA against circ_0092314, and of AsPC-1 cells transfected with circ_0092314 overexpression plasmid (**Figures 2E, F**). Compared with tumors derived from the control cells, those derived from circ_0092314 siRNA-transfected PaCa-2 cells grew slowly (**Figure 2E**). Moreover, overexpression of circ_0092314 significantly increased tumor growth (**Figure 2F**). The results showed that the tumor volume of xenograft in circ_0092314 knockdown group was obviously smaller than the control group (**Figure 2G**). The weight of tumors was lighter in circ_0092314 knockdown group than the control group, consistently (**Figure 2H**). Conversely, the tumor volume and weight were significantly increased in the circ_0092314 overexpressed group (**Figures 2G, H**). These results confirmed the promoting effects of circ_0092314 on PAAD proliferation.

### Circ_0092314 Induces EMT in PAAD Cells

**Figure 3:**
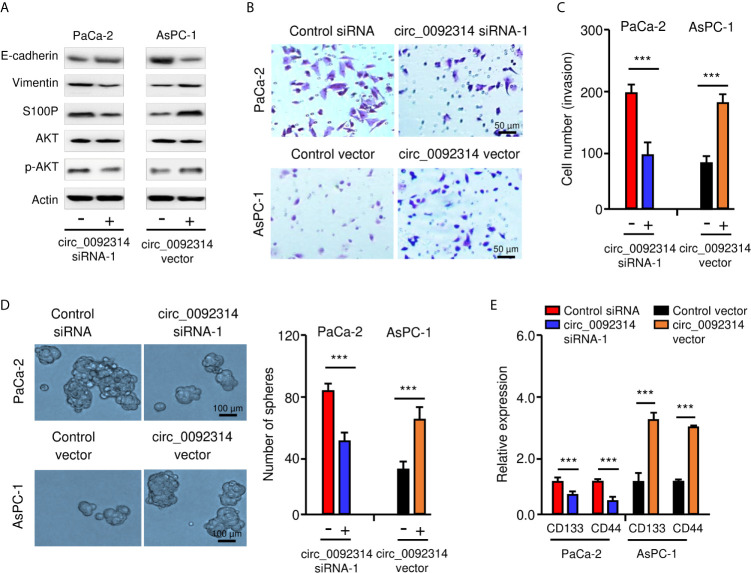
Circ_0092314 induces EMT in PAAD cells. **(A)** Western blotting analysis of the indicated proteins in circ_0092314-silenced or circ_0092314-overexpressing PAAD cells. **(B, C)** Images of representative invasive PAAD cells are shown (**B**), and quantification analysis is presented **(C)**. **(D)** Tumor sphere formation assay for circ_0092314-silenced or circ_0092314-overexpressing PAAD cells, shown by representative images (left) and by quantification (right). **(E)** qRT-PCR assay of CD133 and CD44 expression in circ_0092314-silenced or circ_0092314-overexpressing PAAD cells. ***P < 0.001.

EMT is a critical process that enhances the aggressive properties of cancer cells (2). Therefore, we investigated whether circ_0092314 could promote the EMT features of PAAD cells. Western blotting analysis showed that the expression of E-cadherin was increased, whereas the expression of Vimentin was decreased in PaCa-2 cells after the knockdown of circ_0092314 (**Figure 3A**). In contrast, overexpression of circ_0092314 led to downregulation of E-cadherin and upregulation of Vimentin (**Figure 3A**). Furthermore, we found that knockdown of circ_0092314 could significantly inhibit the invasive abilities and sphere-formation of PaCa-2 cells (**Figures 3B–D**). However, overexpression of circ_0092314 in AsPC-1 cells increased invasive abilities and sphere-forming abilities (**Figures 3B–D**). Our qRT-PCR assay confirmed the downregulated expression of CD133 and CD44 in circ_0092314-silenced PaCa-2 cells, and the upregulated expression of CD133 and CD44 in circ_0092314-overexpressing AsPC-1 cells (**Figure 3E**). These findings indicated that in circ_0092314 promotes EMT and CSC properties of PAAD cells.

### Circ_0092314 Binds to miR-671 and Suppresses Its Expression in PAAD Cells

**Figure 4:**
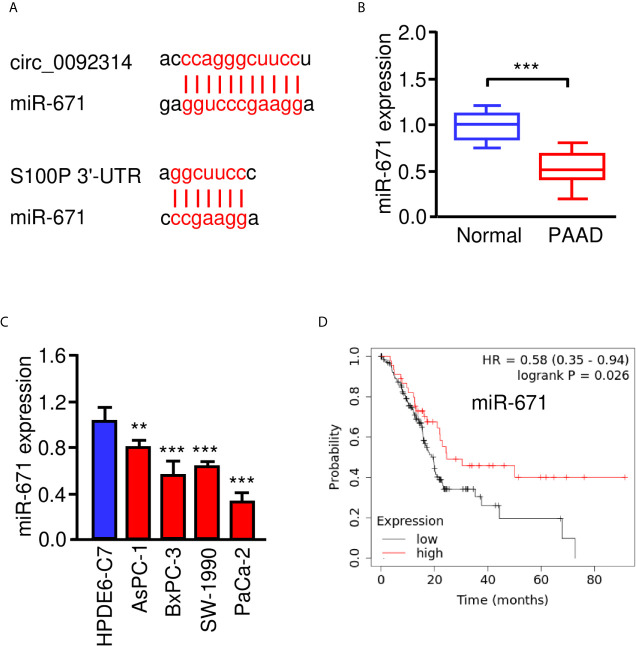
MiR-671 is downregulated in PAAD tissues and positively correlates with patient prognosis. **(A)** The predicted binding site of miR-671 within circ_0092314 and the *S100P* 3′-UTR sequence were shown. **(B)** The expression of miR-671 in PAAD tissues and adjacent normal tissues. **(C)** qRT-PCR analysis of miR-671 expression in four PAAD cell lines and normal pancreatic cells. **(D)** Kaplan-Meier curves for the overall survival of PAAD patients with high or low levels of miR-671 (KM Plotter database). **P < 0.01, ***P < 0.001.

According to the CircInteractome prediction database, a complementary sequence in circ_0092314 for miR-671 was found (**Figure 4A**). The qRT-PCR assay was performed to study the expression of miR-671 in PAAD tissues and adjacent normal tissues. MiR-671 was expressed at lower levels in PAAD tissues compared with normal tissues (**Figure 4B**). The expression of miR-671 in four PAAD cell lines was significantly decreased when compared with HPDE6-C7 cells (**Figure 4C**). The survival curves from Kaplan-Meier Plotter database demonstrated that low level of miR-671 predicted poor outcome in PAAD patients (**Figure 4D**).

**Figure 5:**
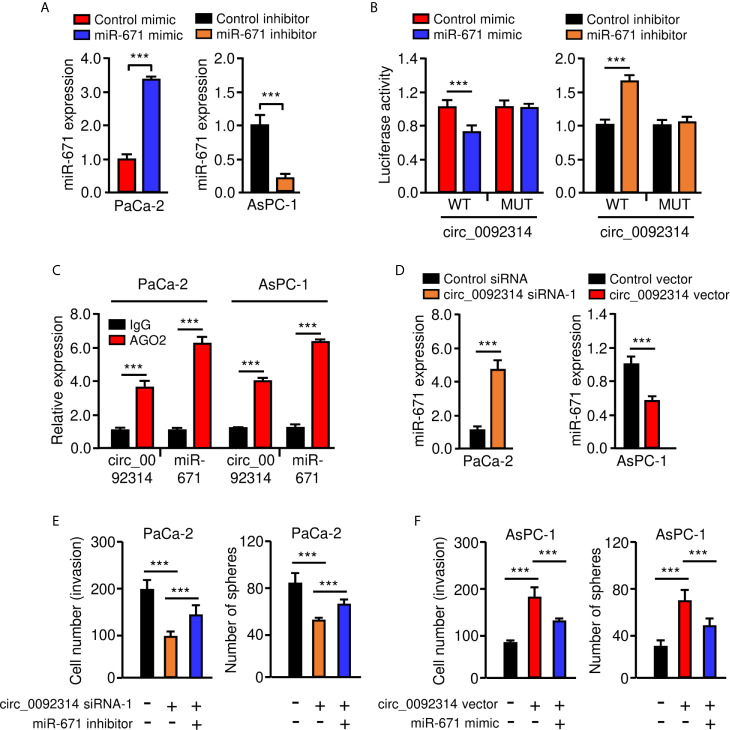
Circ_0092314 binds to miR-671 and suppresses its expression in PAAD cells. **(A)** qRT-PCR analysis of miR-671 in PAAD cells following overexpression or knockdown of miR-671. **(B)** The activities of circ_0092314 reporter containing wild-type (WT) or mutant (MUT) binding sites were determined using luciferase assay following co-transfection with miR-671 mimic or miR-671 inhibitor. **(C)** RIP assay was performed in PaCa-2 or AsPC-1 cells. The levels of circ_0092314 and miR-671 were detected by qRT-PCR assay. **(D)** The expression of miR-671 in PAAD cells following knockdown or overexpression of circ_0092314. **(E)** Cell invasion (left) and sphere formation (right) assay in PaCa-2 cells transfected with (or without) circ_0092314 siRNA, along with (or without) miR-671 inhibitor. **(F)** Cell invasion (left) and sphere formation (right) assay in AsPC-1 cells transfected with (or without) circ_0092314 vector, along with (or without) miR-671 mimic. ***P < 0.001.

Then, a dual-luciferase reporter assay was conducted to confirm the interaction between circ_0092314 and miR-671. The results suggested that transfection with miR-671 mimic significantly attenuated the activity of luciferase reporter containing wild-type (WT) circ_0092314 fragment compared with control mimic, while transfection of miR-671 mimic failed to affect the activity of luciferase reporter containing mutant (MUT) circ_0092314 fragment (**Figures 5A, B**). Transfection with miR-671 inhibitor significantly increased the luciferase activity of the WT circ_0092314 fragment, but not the MUT circ_0092314 fragment (**Figures 5A, B**). RIP experiments suggested that circ_0092314 and miR-671 were preferentially enriched in anti-AGO2-containing complexes compared with anti-IgG (**Figure 5C**). In addition, silencing of circ_0092314 can upregulate the expression of miR-671 in PaCa-2 cells, and overexpression of circ_0092314 in AsPC-1 cells can reduce the levels of miR-671 (**Figure 5D**). Our rescue experiments suggested that knockdown of circ_0092314 suppressed the invasion and sphere formation in PaCa-2 cells, however co-expression of miR-671 inhibitor could abolish these effects of circ_0092314 knockdown (**Figure 5E**). Also, we observed that overexpression of circ_0092314 induced cell invasion and sphere formation, and co-transfection with miR-671 mimic significantly reversed these effects (**Figure 5F**). Collectively, these data demonstrated that circ_0092314 acts as a sponge of miR-761 and suppresses its expression in PAAD cells.

### MiR-671 Suppresses EMT and PAAD Cell Invasion by Inhibiting S100P Expression

**Figure 6:**
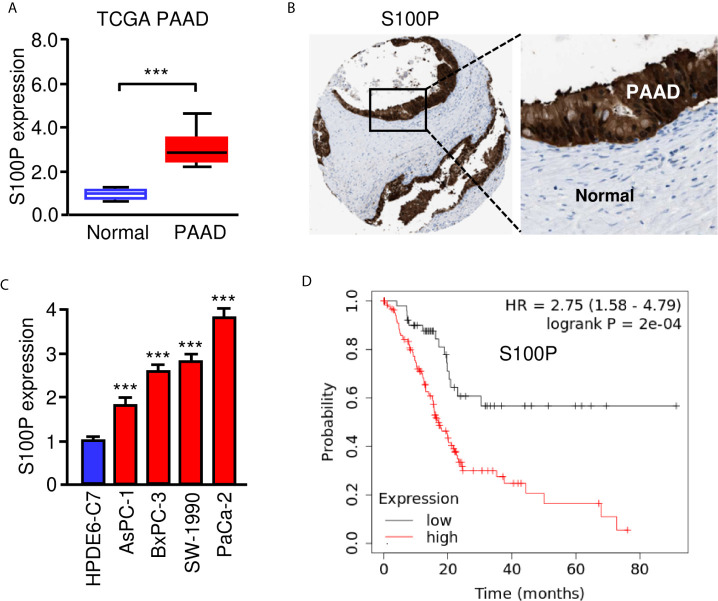
High S100P expression predicts poor prognosis in PAAD. **(A)** qRT-PCR analysis of S100P mRNA expression in PAAD tissues and adjacent normal tissues. **(B)** Immunohistochemical data was downloaded from the Human Protein Atlas database. The staining pattern for S100P protein in PAAD tissues and adjacent normal tissues were shown. **(C)** S100P mRNA expression in PAAD cells and HPDE6-C7 cells. **(D)** Kaplan-Meier curves for the overall survival of PAAD patients with high or low S100P expression (KM Plotter database). ***P < 0.001.

**Figure 7:**
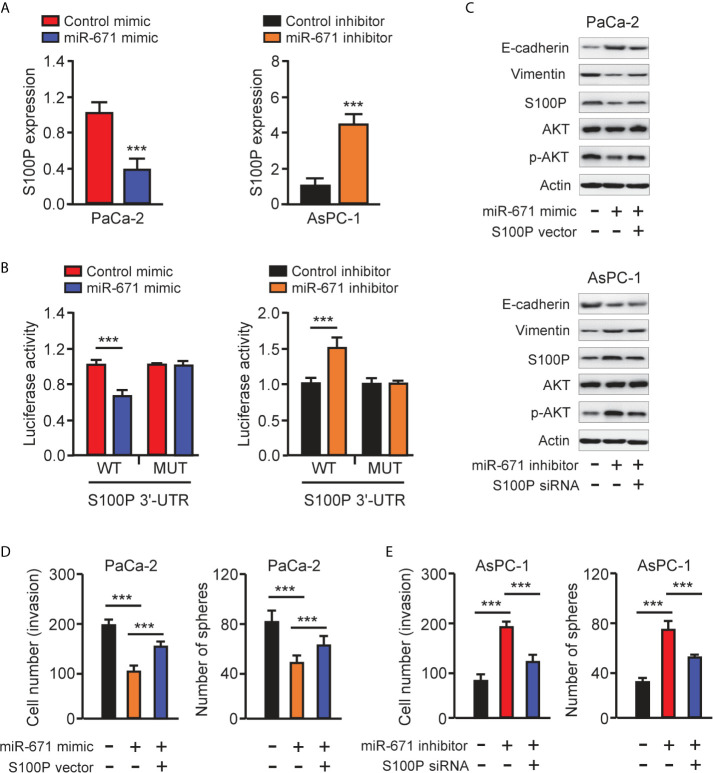
MiR-671 suppresses EMT *via* targeting S100P. **(A)** The levels of S100P mRNA in PAAD cells transfected with miR-671 mimic or miR-671 inhibitor, respectively. **(B)** The activities of S100P 3′-UTR reporter containing WT or MUT miR-671 binding sites were determined using luciferase assay following co-transfection with miR-671 mimic or miR-671 inhibitor. **(C)** Western blotting analysis of the indicated proteins in PAAD cells transfected as indicated. **(D, E)** Cell invasion assay (left) and tumor sphere formation assay (right) in PaCa-2 cells transfected with (or without) miR-671 mimic, with (or without) S100P expression vector **(D)**, and in AsPC-1 cells transfected with (or without) miR-671 inhibitor, with (or without) S100P siRNA **(E)**. ***P < 0.001.

We used the TargetScan database to predict the potential targets of miR-671 and found that S100P was one of the targets of miR-671 (**Figure 4A**). Then, our qRT-PCR results showed the upregulation of S100P in PAAD tissues compared with normal tissues (**Figure 6A**). The protein expression of S100P was further validated in the Human Protein Atlas database (https://www.proteinatlas.org/). The results indicated that the expression levels of S100P were strongly expressed in PAAD tissues, however S100P protein expression was undetectable in adjacent normal tissues (**Figure 6B**). Consistent with these results, the mRNA expression of S100P was highly expressed in PAAD cells than that in HPDE6-C7 cells (**Figure 6C**). Kaplan-Meier survival curves suggested that high levels of S100P expression were associated with a significantly unfavorable overall survival (**Figure 6D**). In order to explore whether miR-671 could directly bind to the 3′-UTR of S100P mRNA, we performed the luciferase reporter assay. Our results showed that overexpression of miR-671 remarkably reduced the luciferase activity of WT S100P 3′-UTR, but had no significant effect on the mutant one (**Figures 7A, B**). Alternatively, inhibition of miR-671 increased the luciferase activity of WT S100P 3′-UTR, without affecting the MUT S100P 3′-UTR (**Figures 7A, B**).

S100P was known to promote EMT in colorectal cancer cells by activating the AKT pathway (7). In line with this finding, our western blotting analysis indicated that miR-671 overexpression led to the downregulation of S100P, Vimentin, and inactivation of AKT signaling, but the upregulation of E-cadherin in PaCa-2 cells (**Figure 7C**). By contrast, inhibition of miR-671 in AsPC-1 cells led to suppression of E-cadherin, induction of Vimentin, and activation of the AKT pathway (**Figure 7C**).

We further examined the contribution of S100P in miR-671-dependent EMT by performing rescue experiments. Remarkably, the ability of miR-671 in suppressing EMT as presented by decreased cell invasion/sphere formation, upregulation of E-cadherin, downregulation of Vimentin, as well as inactivation of the AKT pathway, was largely reversed by overexpression of S100P (**Figures 7C–E**). Conversely, miR-671 inhibitor-induced EMT properties were partially abrogated by knockdown of S100P (**Figures 7C–E**). These data implicated that miR-671 behave as an EMT suppressor in PAAD by targeting S100P and probably by repressing the AKT pathway. Overexpression of circ_0092314 induced S100P expression and activated the AKT pathway in AsPC-1 cells (**Figure 3A**). Consistently, S100P was decreased, and the activity of the AKT pathway was suppressed following circ_0092314 knockdown in PaCa-2 cells (**Figure 3A**).

**Figure 8:**
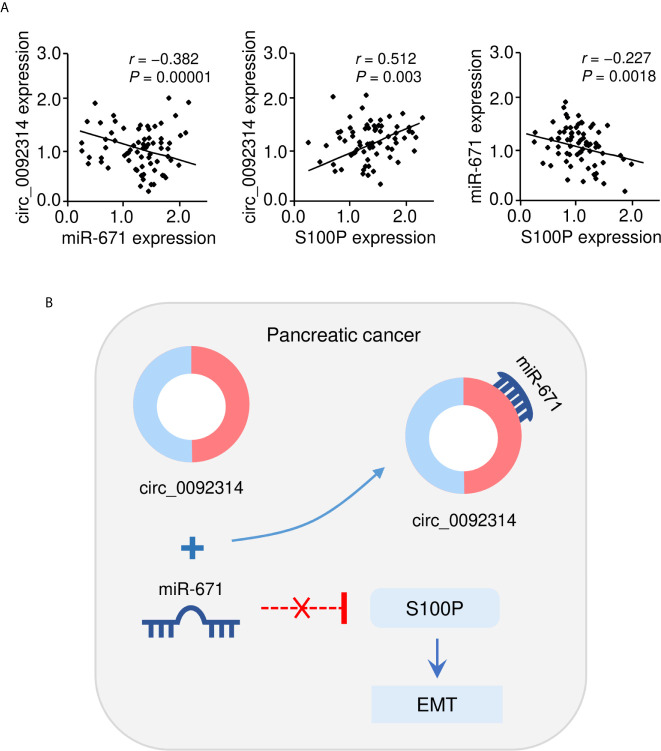
The correlation between circ_0092314 and miR-671/S100P expression in PAAD tissues. **(A)** The correlation between circ_0092314 expression and miR-671/S100P expression in PAAD tissues was examined using qRT-PCR assay. **(B)** A proposed mechanistic model in which circ_0092314 can sponge miR-671 to increase S100P expression, thereby promoting EMT and PAAD cell invasion.

Furthermore, Pearson correlation analysis indicated that the expression of circ_0092314 was negatively correlated with miR-671 expression, and positively correlated with S100P mRNA expression in PAAD cancer tissues (**Figure 8A**). We also observed that there was a negative correlation between miR-671 expression and S100P mRNA expression in PAAD cancer tissues (**Figure 8A**). Overall, in this study, we identified circ_0092314, as a novel oncogenic circRNA induces EMT and invasion of PAAD cells *via* elevating the abundance of S100P by sponging a tumor suppressor miR-671 (**Figure 8B**).

## Discussion

The traditional prognostic markers for PAAD include histological subtype, vascular and perineural invasion, the presence of desmoplastic reaction, tumor budding and EMT (15). In addition, new and emerging prognostic biomarkers, including miRNA, long non-coding RNA and recently circRNA, have been reported (15, 16). For instance, circ-LDLRAD3 was significantly upregulated in PAAD tissues and plasma, and a high level of circ-LDLRAD3 was positively associated with tumor venous invasion and lymphatic metastasis (17). Interestingly, circ-LDLRAD3 combined with CA19-9 was confirmed to have higher sensitivity and specificity for the diagnosis of PAAD (17). In addition, high expression of circ_0030235 was an independent prognostic indicator of unfavorable overall survival for PAAD patients according to a multivariate Cox analysis (18). Here, we provided new evidence to show that the expression of circ_0092314 was significantly higher in clinical PAAD tissues than in adjacent normal tissues, and its expression was closely associated with aggressive behavior of PAAD. Importantly, we have shown that those PAAD patients with high circ_0092314 expression had worse clinical outcomes, indicating the potential of circ_0092314 as a promising prognostic biomarker for PAAD patients. Whether its expression was changed in the plasma of patients with PAAD, whether circ_0092314 expression is correlated with CA19-9 levels, deserve further investigation.

Growing studies have supported the existence of complex interactions between circRNAs and miRNAs in PAAD cells (19, 20), in which circRNAs regulates the expression and activity of miRNAs by competitively binding their target sites on protein-coding mRNAs. Here, we showed for the first time that circ_0092314 promotes EMT features by binding to miR-671 to induce the expression of S100P. Our bioinformatic analysis using the CircInteractome database has shown that circ_0092314 hosts multiple binding sites for diverse miRNAs and RNA-binding proteins (data not shown), suggesting that circ_0092314 probably act as sponges for different cytoplasmic miRNAs, or interact with RNA-binding proteins to generate RNA-protein complexes that can also control the EMT process and PAAD metastasis.

Over the last several years, although we should acknowledge that numerous efforts to improve the efficacy of surgery and chemotherapy in PAAD patients have been made, there are still few reliable biomarkers or effective therapeutic strategies for daily clinical practice in PAAD. Increasing evidence indicated that circRNAs have great potential to regulate cancer cell proliferation, apoptosis, invasion, EMT, metastasis and response to chemotherapy, implying that circRNAs may be used as novel potential therapeutic targets for treating various tumors including PAAD (9, 10, 21, 22). Here, we confirmed that the knockdown of circ_0092314 suppressed the growth and invasion of PAAD cells *in vitro*, and resulted in reduced tumor size and tumor weight *in vivo*, suggesting that targeting circ_0092314 is a potential therapeutic strategy for PAAD.

Downregulation of miR-671 has been observed in a panel of human tumors, such as osteosarcoma (23), prostate cancer (24), lung cancer (25), breast cancer (26), and gastric cancer (27). In these tumors, miR-671 was demonstrated to show inhibit the malignant phenotypes of tumor cells by targeting SOX6, CCND2, and FOXM1 (23–27). However, miR-671 may function as an oncogenic miRNA in other types of cancer (28, 29). In our study, miR-671 was identified as a downstream effector of circ_0092314 in PAAD cells, in which the tumor-promoting effects of circ_0092314 could be blocked through miR-671 overexpression. This ability of miR-671 to suppress the EMT properties of PAAD cells provides insights into the molecular mechanisms of PAAD metastasis and a path for inhibiting the metastatic spread of PAAD or other cancers. The detailed mechanisms by which miR-671 mediates EMT and PAAD cell invasion deserve future studies.

S100P, a calcium-binding protein, can advance tumor progression and metastasis in pancreatic and several other cancers (6, 30, 31). S100P has previously been demonstrated to regulate the proliferation, migratory and invasive capabilities of PAAD cells (6). In addition, decreased PAAD growth was observed following S100P silencing in an orthotropic mouse model (6). S100P promotes EMT, migration and invasion of colon cancer cells by up-regulating S100A4 through AKT activation (7). Consistent with these reports, we found that S100P acts as an important EMT activator in PAAD cells, and its oncogenic functions might be involved in the activation of the AKT pathway. Together, our results suggest that dysregulation of the circ_0092314/miR-671/S100P axis is responsible for PAAD progression, and these molecules are potential therapeutic targets for suppressing the EMT in metastatic PAAD.

In summary, our findings support the idea that the circ_0092314/miR-671/S100P signaling plays crucial roles in regulating EMT phenotypes of PAAD cells and suggest that this signaling pathway might be an effective target for PAAD therapy.

## Data Availability Statement

The original contributions presented in the study are included in the article/supplementary material. Further inquiries can be directed to the corresponding author.

## Ethics Statement

The studies involving human participants were reviewed and approved by The Research Ethics Committee of Tongji Hospital, Tongji Medical College, Huazhong University of Science and Technology, China. The patients/participants provided their written informed consent to participate in this study. The animal study was reviewed and approved by The Institutional Animal Care and Use Committee of Tongji Hospital, Tongji Medical College, Huazhong University of Science and Technology, China.

## Author Contributions 

QF designed the experiments. QS, GZ, YZ, and JT performed the experiments. SX, HG, and XZ made significant revisions to the manuscript. All authors contributed to the article and approved the submitted version.

## Funding 

This study was supported by the National Natural Science Foundation of China (No. 81974381).

## Conflict of Interest

The authors declare that the research was conducted in the absence of any commercial or financial relationships that could be construed as a potential conflict of interest.
